# Optimized Synthesis of New N-Mustards Based on 2-Mercaptobenzoxazole Derivatives with Antitumor Activity

**DOI:** 10.3390/biomedicines9050476

**Published:** 2021-04-26

**Authors:** Corina Cheptea, Valeriu Sunel, Ana Cezarina Morosanu, Dan Gheorghe Dimitriu, Mihaela Maria Dulcescu-Oprea, Mihai-Daniel Angheluta, Mihaela Miron, Cristina Delia Nechifor, Dana Ortansa Dorohoi, Razvan Nicolae Malancus

**Affiliations:** 1 Department of Biomedical Sciences, Faculty of Biomedical Engineering, “Grigore T. Popa” University of Medicine and Pharmacy, 700115 Iasi, Romania; corina.cheptea@umfiasi.ro; 2 Faculty of Chemistry, Alexandru Ioan Cuza University, 700506 Iasi, Romania; vsunel@uaic.ro; 3 Faculty of Physics, Alexandru Ioan Cuza University, 700506 Iasi, Romania; cezarina_morosanu@yahoo.com (A.C.M.); ddorohoi@uaic.ro (D.O.D.); 4 Regional Institute of Oncology, 700483 Iasi, Romania; opreamihaelamaria@yahoo.com; 5 Faculty of Medicine, “Iuliu Hateganu” University of Medicine and Pharmacy, 400012 Cluj-Napoca, Romania; mihai.daniel.angheluta@gmail.com; 6 Faculty of Medicine, “Grigore T. Popa” University of Medicine and Pharmacy, 700115 Iasi, Romania; mihaela-miron@email.umfiasi.ro; 7 Department of Physics, Faculty of Machine Manufacturing and Industrial Management, 700050 Iasi, Romania; cd13_nechifor@yahoo.com; 8 Department of Physiology and Pathophysiology, Faculty of Veterinary Medicine, “Ion Ionescu de la Brad” University of Agricultural Sciences and Veterinary Medicine, 700490 Iasi, Romania; razvanmalancus@uaiasi.ro

**Keywords:** 2-mercaptobenzoxazole, N-mustards, anti-inflammatory activity

## Abstract

New di-(β-chloroethyl)-amides of some acids derived from 2-mercaptobenzoxazole were prepared by reaction of the corresponding pivalic mixed anhydrides with di-(β-chloroethyl)-amine. A study regarding the optimization of the chemical reactions was made for the case of di-(β-chloroethyl)-amines. The quantum chemical analysis by Spartan’14 was made in order to establish the most stable configuration of the ground electronic states for the obtained chemical structures and some physico-chemical parameters of N-mustards reported in this paper. Mercaptobenzoxazoles substituted in the side chain with the cytotoxic group show antitumor activity and they inhibit *Ehrlich Ascites* in an appreciable proportion compared to the drug I.O.B.-82, as our studies evidenced.

## 1. Introduction

Cancer can be considered one of the most pressing concerns of research in medicine, chemotherapy, phytochemistry, and biology. Chemotherapy alone or in combination with radio-immunosurgical treatment is the major strategy in the fight against cancer.

In addition to drugs that support the current possibilities of clinical treatment, the range of antitumor compounds is constantly growing [1,2,3,4,5,6,7,8,9,10].

However, there are some drawbacks in the administration of various chemicals used as cytostatic drugs. First, their poor selectivity towards cancer cells, they also act on normal cells and are, therefore, toxic for the body. Second, there are concerns about the installation of the resistance of malignant tumors against the used drugs [11]. Nowadays, the most active and widely used class of cytostatics is that of alkylating substances. The cytostatics containing the di-(β-chloroethyl)-amine group in their structure have the widest utility.

When the active group is attached to a radical of an aliphatic hydrocarbon, those N-mustards have a reduced selective action against cancer cells and a high toxicity to the normal cells [12].

N-mustards chemotherapy has taken a step forward in the use of aromatic and heterocyclic di-(β-chloroethyl)-amines. Their lower toxicity is due to the action of the aromatic or heterocyclic support, which causes a decrease in the basicity of the nitrogen atoms and the rate of alkylation, respectively. The main mode of action of di-(β-chloroethyl)-amines consists in the possibility of an intramolecular cyclization with the formation of an immonium cation consisting of an ethylene ring of three atoms, which, being unstable, easily breaks and can react through carbon as an alkylating agent for substances possessing active groups: NH_2_, SH, and so forth.

It is assumed that, through the alkylating group, the N-mustards bind to the active groups NH_2_, SH, and so forth, from two protein macromolecules, nucleoproteins, deoxyribonucleic acids that participate in the multiplication process of the neoplastic cells and, by forming cross-links, forms a bridge between two nucleoprotein macromolecules or protein molecules and enzymes (cross-linking), preventing them from participating in accelerated biosynthesis, stopping the anarchic division of malignant cells [13].

The specialized literature offers synthesis techniques for some N-mustards in which, by varying the support of the cytotoxic group, alkylated substances with advantageous chemotherapeutic indices and lower toxicity were obtained [4,5,13,14,15,16,17,18].

Benzoxazole and its derivatives constitute an important class of compounds that possess a wide range of pharmacological properties [19,20,21,22,23,24,25,26,27,28]. In addition, some of the benzoxazoles also show cytotoxic activity against malignant cells [29,30].

The aim of the present study is to propose new N-mustard compounds for biomedical applications in cancer therapy. As new compounds, they are studied both for the optimization of their obtaining reactions and for establishing some characteristics based on quantum mechanical analysis. Their very low toxicity and the results provided after testing on experimental animals recommend them as potential candidates for antitumor drugs development.

## 2. Materials and Methods

The chemical reactants were provided by Merck Company (Darmstadt, Germany) and Fluka (Darmstadt, Germany) and used without any purification.

The purity of the obtained substances was checked by quantitative elemental analysis, as well as Fourier-transform infrared (FT-IR) and nuclear magnetic resonance (NMR) spectroscopy. Quantitative elemental analysis was performed by using the Exeter Analytical CE 440 (Exeter Analytical UK Ltd., Coventry, UK). BRUKER Tensor-27 FT-IR (ATR) spectrophotometer (Bruker Optik GmbH, Ettlingen, Germany) was used to record the FT-IR spectra. ^1^H-NMR spectra (DMSO-d_6_, 400 MHz) were recorded using a BRUKER ARX 400 spectrometer (Bruker BioSpin GmbH, Rheinstetten, Germany) equipped with 5 mm QNP ^1^H/^13^C/^31^P/^19^F samples and Silicon Graphics INDIGO^2^ workstation (Silicon Graphics, Inc., Mountain View, CA, USA).

Statistical models based on the factorial design [31,32,33] were applied to establish the best conditions in which the reaction rates can be maximized. To decide the situations for which the reaction yield does not depend on a given variable, the Student’s *t*-test [32] was applied.

The quantum mechanical analysis was performed by the help of Spartan’14 software (Wavefunction, Inc., Irvine, CA, USA) [34,35], using the density functional method (Density Functional EDF2, 6-31 G*).

The acute toxicity was established by evaluation of produced mortality. The toxicity degree indicates the maximum bearable dose LD_0_, minimum lethal dose LD_100_, and the killing dose that kills 50% of the experimental animals LD_50_, these representing the best clues in the interpretation of the results. In the determination of the acute toxicity of the compounds, mice weighing 20 ± 2 g were used, the experimental groups consisting of 10 animals of both sexes. The substances were administered intraperitoneally as a suspension in Tween 80 (Sigma-Aldrich, Darmstadt, Germany) and the mortality was recorded at 24 h, 48 h, and 7 days. The general condition, the appearance of the hair, the behavior, the appearance of the tremors, and so forth were observed. The Spearman–Kärber method [36] allowed for the quick determination of LD_50_ on a small number of animals, using the next calculation method:

(1) $$LD_{50}=LD_{100}-\sum \frac{a\times b}{n}$$

where *a* is the difference between two successive doses of the administrated substance, *b* is the average number of dead animals in two successive doses, and *n* is the total number of animals in the group.

To study the cytostatic action, the A_2_G mice weighing 25–30 g (±2 g) were used as experimental animals and *Ascita Ehrlich* has been used as an experimental tumor. The transplantation was performed intraperitoneally. The substances were administered as suspensions in 1% methylcellulose, by single injections on the 7th day after transplantation. The concentrations of 400, 200, and 40 mg/kg body weight were used. The experimental groups included 20 animals for each concentration, while the control groups had 10 tumored animals. The inhibition was calculated according to the methods from the literature [37], 7 days after the administration of the substances.

## 3. Results and Discussion

### 3.1. Chemical Reactions

The pharmacodynamic action of various chemicals is the result of their interaction with substances from the biological substrate, under the influence of biochemical factors. Consequently, the synthesis and study of a wide range of chemical compounds that contain bioactive groups in the molecule will allow the establishment of a more accurate correlation between structure and biological activity.

Based on the previous research [13,14,15,16,17,38,39], the synthesis of new N mustard derivatives containing the benzoxazole heterocycle and the di-(β-chloroethyl)-amine group in their molecules was made. Thus, it becomes interesting to study the mutual influence of these components that are present in the same molecule, as well as their contribution to the overall biological activity of the substance.

**Scheme 1:**
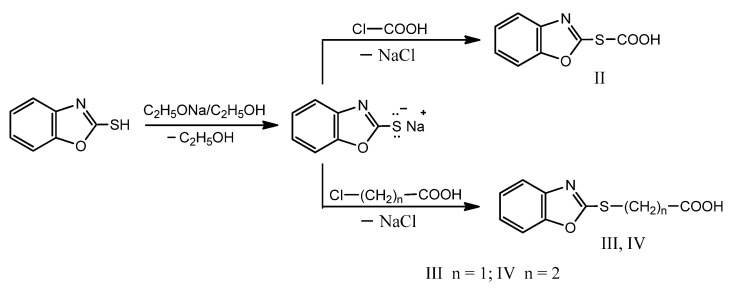
Synthesis of the benzoxazole-2-yl-mercapto-(II–IV) acids.

In our research, we aimed to graft the di-(β-chloroethyl)-amine group in different positions on the side chain of the oxazole ring within the 2-mercapto benzoxazole substrate. For this purpose, by the hot dissolution of 2-mercaptobenzoxazole in alcoholic solution containing sodium ethoxide, the sodium salt of 2-mercaptobenzoxazole (I) was obtained, which, treated in water-acetone solution, with chloroformic acid, chloroacetic acid and β-chloropropionic acid, led to benzoxazole-2-yl-mercapto-formic (II), acetic (III), and β-propionic (IV) acids (Scheme 1).

The synthesis of the compounds I–IV was confirmed by elemental and spectral analysis (FT-IR, ^1^H-NMR).

The IR spectra of the compound (I) show a characteristic band of the aromatic ring at 3000 cm^−1^. The band attributed to the valence vibration of the cyclic C = N group can be noticed at 1481 cm^−1^. The C–S group gives absorption at 685 cm^−1^. In the ^1^H-NMR spectra, the signal of the aromatic protons appears in the form of a doublet in the range 6.94–7.47 ppm.

The IR spectra of the derivatives (II–IV) show a band at 3356–3368 cm^−1^ corresponding to the valence vibrations of the COOH group and in the immediate proximity, at 2982–3060 cm^−1^, an intense absorption band given by the vibrations of the aromatic CH group. The cyclic C = N group is highlighted by the band at 1638–1644 cm^−1^. The bands specific to the valence vibration of the C–S connection appear at 720–752 cm^−1^. The band at 1076–1080 cm^−1^ is assigned to the cyclic C–O group. In the ^1^H-NMR spectra of the compounds (II-IV), the proton of the COOH group is highlighted at 11.56–11.76 ppm, while signals of the aromatic protons are present at 7.13–7.69 ppm. For compounds (III) and (IV), due to the nucleophilic nature of the COOH group and the two heteroatoms (N and O) of the benzoxazole heterocycle, the peak values for the protons of the CH_2_ group are found close to the protons in the benzene nucleus, namely at 7.6–7.11 ppm.

It is known [13,40,41,42,43,44,45] that the functionalization of di-(β-chloroethyl)-amine is performed in the presence of an inert solvent, using anhydrides, acid chlorides or other acylating agents. The use of the mixed anhydride method [46] allowed us to functionalize the di-(β-chloroethyl)-amine with acids derived from 2-mercaptobenzoxazole and to obtain, with good yield, some di-(β-chloroethyl)-new benzoxazole amides.

**Scheme 2:**
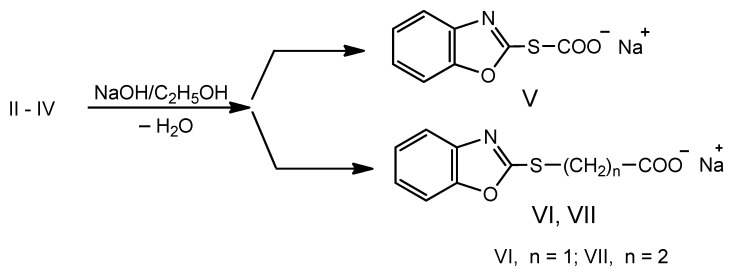
Synthesis of the sodium salts of benzoxazol-2-yl-mercapto-formic, acetic, and β-propionic acids (V–VII), respectively.

Initially, the sodium salts of the benzoxazole-2-yl-mercapto-formic, acetic, and β-propionic (V–VII) acids were synthesized by refluxing a mixture of acid (II–IV) in alcoholic solution with sodium hydroxide (Scheme 2).

The structure of the compounds V-VII was also investigated by elemental and spectral analysis (FT-IR, ^1^H-NMR). The IR spectra for the compounds V-VII show absorption at 1608–1640 cm^−1^, specific to the COO^–^ group. The ^1^H-NMR spectra of the compounds V-VII show the aromatic protons as a doublet at 7.12–7.68 ppm and the protons of the CH_2_ group (in the compounds VI and VII) as a multiplet in the region of 7.05–7.12 ppm.

**Scheme 3:**
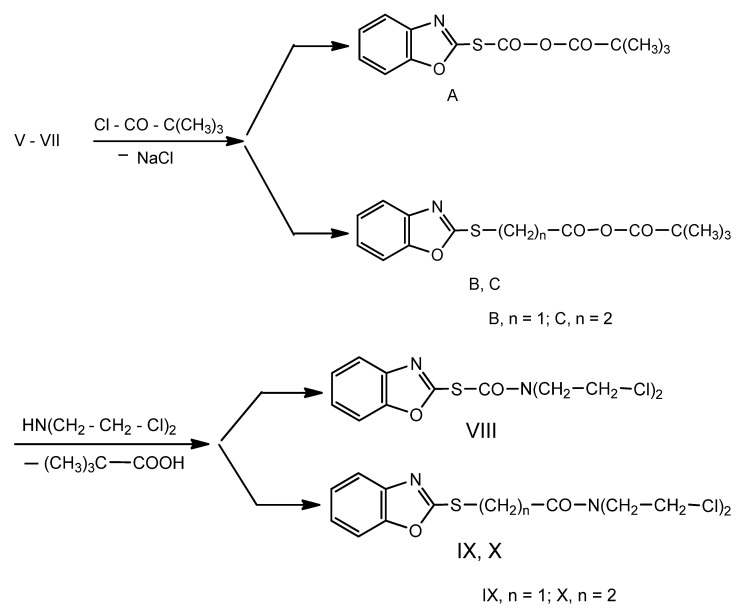
Reactions for obtaining di-(β-chloroethyl)-amides of benzoxazole-2-yl-mercapto-formic (VIII), acetic (IX) and β-propionic (X) acids, respectively.

In order to increase the reactivity of the carboxyl group in sodium salts (V–VII), they were converted by treatment with pivaloyl chloride (trimethylacetic acid chloride) to mixed anhydrides (A–C), capable of reacting rapidly and under mild conditions with di-(β-chloroethyl)-amine. The mixed anhydrides (A–C) are unstable and it is difficult to isolate them, so we chose for their in situ use a dichloromethine solution in which di-(β-chloroethyl)-amine is dissolved as a free base. The reaction mixture was stirred for three hours, filtered, and recrystallized from the anhydrous dioxane precipitate to give N-mustards (VIII-X) (Scheme 3).

The structure of the compounds VIII–X was established by elemental and spectral analysis (FT-IR, ^1^H-NMR). The IR spectra show characteristic bands of the three N-mustards. In the area 3094–3097 cm^−1^, there are the bands corresponding to the vibration of the aromatic CH group. The C = O amide bond produces a well-highlighted band at 1623 cm^−1^ and 1624 cm^−1^. The vibration of the C = N group appears at 1490–1493 cm^−1^. In the IR absorption spectra of N-mustards VIII-X, the C–N stretching frequency was identified at 1283–1306 cm^−1^, while the C–Cl stretching frequency was identified between 747–789 cm^−1^. In the ^1^H-NMR spectra of the compounds VIII–X, multiplets between δ = 3.16–3.41 ppm (VIII), δ = 3.55–3.57 ppm (IX) and δ = 2.55–3.39 ppm, respectively, whose integrated surface satisfactorily coincides, in all cases, with the eight existing protons, were obtained for the protons from the N-mustard group.

### 3.2. Experimental Procedure

#### 3.2.1. Sodium Salt of the 2-Mercaptobenzoxazole (I)

First, 0.2 mol 2-mercaptobenzoxazole were treated with 0.2 mol metal sodium dissolved in 120 mL anhydrous ethyl alcohol. The obtained solution was refluxed for 90 min on a water bath, then hot filtered to remove any impurities. Excess of alcohol was removed by distillation under reduced pressure, then, after cooling, the precipitate was separated, filtered in vacuum, and then dried.

Light gray solid (32.76 g; yield 95%); M.p. = 298–300 °C.

Elemental analysis calculated for C_7_H_4_NOSNa (%): C, 48.55; H, 2.31; N, 8.09; S, 18.49. Found: (%): C, 48.67; H, 2.65; N, 8.31; S, 18.90.

IR; ν_max_ (cm^−1^): 3000 (CHAr); 1481 (C = N); 685 (C–S).

^1^H-NMR (DMSO-d_6_, 400 MHz), δ (ppm): 6.94–7.14 (d, 2H, CHAr); 7.31–7.47 (d, 2H, CHAr).

#### 3.2.2. Benzoxazole-2-Yl-Mercapto-Formic, Acetic, β-Propionic Acids, Respectively (II–IV)

Next, 0.05 mol of sodium salt of 2-mercaptobenzoxazole (I) were dissolved in 60 mL of water-acetone (1:1) to give a clear gray solution. Then, 0.05 mol of acid (chloroformic, chloroacetic and β-chloropropionic) were added in portions, under vigorous stirring. The reaction mixture was heated at 40–45 °C, stirring for 100–120 min. Towards the end, an abundant white-yellow precipitate appeared instantly. It was filtered by vacuum, dried, and purified by recrystallization from boiling water.

#### 3.2.3. Benzoxazole-2-Yl-Mercapto-Formic Acid (II)

White, crystalline solid (6.24 g; yield, 64%); M.p. = 111–112 °C.

Elemental analysis calculated for C_8_H_5_NO_3_S (%): C, 49.23; H, 2.56; N, 7.17; S, 16.41. Found: (%): C, 49.57; H, 2.78; N, 7.36; S, 16.68.

IR; ν_max_ (cm^–1^): 2988 (CHAr); 1644 (C = N); 1076 (C–O); 720 (C–S); 3360 (COOH).

^1^H-NMR (DMSO-d_6_, 400 MHz), δ (ppm): 7.26–7.39 (d, 2H, CHAr); 7.41–7.69 (d, 2H, CHAr); 11.76 (s, 1 H, COOH).

#### 3.2.4. Benzoxazole-2-YL-Mercapto-Acetic Acid (III)

White, crystalline solid (8.04 g; yield, 77%); M.p. = 113–114 °C.

Elemental analysis calculated for C_9_H_7_NO_3_S (%): C, 51.67; H, 3.34; N, 6.69; S, 15.31. Found: (%): C, 52.02; H, 3.51; N, 6.97; S, 15.64.

IR; ν_max_ (cm^–1^): 2982 (CHAr); 1641 (C = N); 1076 (C–O); 760 (C–S); 784 (–CH_2_–S); 3356 (COOH).

^1^H-NMR (DMSO-d_6_, 400 MHz), δ (ppm): 7.06–7.08 (m, 2H, CH2); 7.13–7.16 (d, 2H, CHAr); 7.24–7.32 (d, 2H, CHAr); 11.60 (s, 1 H, COOH).

#### 3.2.5. Benzoxazole-2-Yl-Mercapto-β-Propionic Acid (IV)

White, crystalline solid (7024 g; yield, 63%); M.p. = 120–122 °C.

Elemental analysis calculated for C_10_H_9_NO_3_S (%): C, 53.01; H, 4.03; N, 6.27; S, 14.34. Found: (%): C, 53.35; H, 4.22; N, 6.65; S, 14.61.

IR; ν_max_ (cm^–1^): 3060 (CHAr); 1638 (C = N); 1080 (C–O); 752 (C–S); 788 (–CH2–S); 3360 (COOH).

^1^H-NMR (DMSO-d_6_, 400 MHz), δ (ppm): 7.09–7.11 (m, 4H, CH_2_); 7.27–7.28 (d, 2H, CHAr); 7.60 (d, 2H, CHAr); 11.51 (s, 1 H, COOH).

#### 3.2.6. Sodium Salts of Benzoxazole-2-Yl-Mercapto-Formic, Acetic, β-Propionic Acids (V–VII)

0.02 moles of sodium hydroxide p.a. are introduced into 100 mL of anhydrous ethyl alcohol with 0.02 moles of acid (II–IV). The mixture is stirred until the acids are completely dissolved, then is refluxed for 60 min. The alcoholic solution is filtered and then concentrated by distillation under reduced pressure to a volume of 25–30 mL. The content of the flask still hot is transferred to a crystallizer in which, by cooling, the sodium salt (V–VII) precipitates in the form of crystals in an abundant layer.

#### 3.2.7. Sodium Salt of Benzoxazole-2-Yl-Mercapto-Formic Acid (V)

White, crystalline solid (4.07 g; yield, 94%); M.p. = 188–190 °C.

Elemental analysis calculated for C_8_H_4_NO_3_SNa (%): C, 44.23; H, 1.84; N, 6.45; S, 14.74. Found: (%): C, 44.38; H, 2.07; N, 6.78; S, 15.03.

IR; ν_max_ (cm^–1^): 720 (C–S); 1610 (COO–).

^1^H-NMR (DMSO-d_6_, 400 MHz), δ (ppm): 7.25–7.68 (d, 2H, CHAr).

#### 3.2.8. Sodium Salt of Benzoxazole-2-Yl-Mercapto-Acetic Acid (VI)

White, crystalline solid (4.48 g; yield, 97%); M.p. = 206–208 °C.

Elemental analysis calculated for C_9_H_6_NO_3_SNa (%): C, 46.75; H, 2.59; N, 6.06; S, 13.85. Found: (%): C, 46.96; H, 2.77; N, 6.43; S, 14.18.

IR; ν_max_ (cm^–1^): 784 (S–CH2); 1608 (COO–).

^1^H-NMR (DMSO-d_6_, 400 MHz), δ (ppm): 7.05–7.07 (m, 2H, CH_2_); 7.12–7.33 (d, 2H, CHAr).

#### 3.2.9. Sodium Salt of Benzoxazole-2-Yl-Mercapto-β-Propionic Acid (VII)

White, crystalline solid (4.50 g; yield, 92%); M.p. = 209–221 °C.

Elemental analysis calculated for C_10_H_8_NO_3_SNa (%): C, 48.97; H, 3.26; N, 5.71; S, 13.16. Found: (%): C, 49.28; H, 3.43; N, 6.04; S, 13.34.

IR; ν_max_ (cm^–1^): 790 (–CH_2_–S); 1610 (COO^–^).

^1^H-NMR (DMSO-d_6_, 400 MHz), δ (ppm): 7.08–7.12 (m, 4H, 2CH_2_); 7.26–7.62 (d, 2H, CHAr).

#### 3.2.10. Di-(β-Chloroethyl)-Amides of the Benzoxazole-2-Yl-Mercapto-Formic, Acetic, β-Propionic Acids (VIII–X)

First, 0.02 mol of sodium salt (V–VII) were dissolved in 50 mL of anhydrous dichloromethane, cooled to 3–5 °C and, under stirring, 0.02 mol of freshly distilled pivalic chloride was added. The mixed anhydride formed was treated with 0.025 moles of di-(β-chloroethyl)-amine and then the stirring at 10–12 °C for 60 min was continued. Interrupting the cooling, the reaction mixture was stirred for 3 h, filtered, and recrystallized from the anhydrous dioxane precipitate. N-Mustards (VIII–X) were obtained.

#### 3.2.11. Di-(β-Chloroethyl)-Amide of Benzoxazole-2-Yl-Mercapto-Formic Acid (VIII)

Solid white-yellow, crystalline (4.59 g; yield, 72%); M.p. = 195–197 °C.

Elemental analysis calculated for C_12_H_12_N_2_O_2_SCl_2_ (%): C, 45.14; H, 3.76; N, 8.77; S, 10.03; Cl, 22.25. Found: (%): C, 45.31; H, 3.98; N, 8.98; S, 10.37; Cl, 22.56.

IR; ν_max_ (cm^–1^): 3095 (CHAr); 1491 (C = N); 1305 (C–N tertiary); 1623 (C = O amidic); 748, 789 (C–Cl).

^1^H-NMR (DMSO-d_6_, 400 MHz), δ (ppm): 3.16–3.19 (m, 4H, 2CH_2_); 3.40–3.44 (m, 4H, 2CH_2_); 7.39–7.41 (d, 2H, CHAr); 7.71–7.73 (d, 2H, CHAr).

#### 3.2.12. Di-(β-Chloroethyl)-Amide of Benzoxazole-2-Yl-Mercapto-Acetic Acid (IX)

Yellow, crystalline solid (5.09 g; yield, 84%); M.p. = 178–180 °C.

Elemental analysis calculated for C_13_H_14_N_2_O_2_SCl_2_ (%): C, 46.84; H, 4.20; N, 8.40; S, 9.60; Cl, 21.32. Found: (%): C, 46.95; H, 4.44; N, 8.74; S, 9.99; Cl, 21.67.

IR; ν_max_ (cm^–1^): 3097 (CHAr); 790 (S–CH2); 1493 (C = N); 1283 (C–N tertiary); 1624 (C = O amidic); 749 (C–Cl).

^1^H-NMR (DMSO-d_6_, 400 MHz), δ (ppm): 3.35–3.37 (m, 4H, 2CH_2_); 3.55–3.57 (m, 4H, 2CH_2_); 4.62 (s, 2H, CH_2_–S); 7.38–7.40 (d, 2H, CHAr); 7.60–7.61 (d, 2H, CHAr).

#### 3.2.13. Di-(β-Chloroethyl)-Amide of Benzoxazole-2-Yl-Mercapto-β-Propionic Acid (X)

White-yellow, crystalline solid (4.71 g; yield, 68%); M.p. = 187–189 °C.

Elemental analysis calculated for C_14_H_16_N_2_O_2_SCl_2_ (%): C, 48.41; H, 4.61; N, 8.06; S, 9.22; Cl, 20.46. Found: (%): C, 48.70; H, 4.96; N, 8.47; S, 9.60; Cl, 20.82.

IR; ν_max_ (cm^–1^): 3094 (CHAr); 785 (S –CH_2_); 1490 (C = N); 1306 (C–N tertiary); 1623 (C = O amidic); 747, 788 (C–Cl).

^1^H-NMR (DMSO-d_6_, 400 MHz), δ (ppm): 2.55–2.57 (m, 2H, CH_2_); 3.37–3.39 (m, 4H, 2CH_2_); 3.60–3.62 (m, 4H, 2CH_2_); 4.32–4.34 (m, 2H, CH_2_–S); 7.71–7.73 (d, 2H, CHAr); 7.85–7.87 (d, 2H, CHAr).

### 3.3. Optimization of the Reactions

In the factorial experiments [31,32,33], the reaction yield is considered as being an indicator of the reaction optimization. It is markedly influenced by some conditions of the chemical reactions as temperature, time interval, solvent, solvent mixture, impurities, or pressure. These parameters are considered real variables. Some of these are significant variables.

The yield of the chemical reactions for obtaining the benzoxazoles derivatives VIII–X depends on two significant variables: temperature and time interval of reactions. Let us consider temperature (*T*, in °C, noted with *X*_1_) and interval of time (*t*, in hours, noted with *X*_2_) as being real variables of the experiment. In order to facilitate the calculations, dimensionless variables *x*_1_ and *x*_2_ are introduced instead of the real variables *X*_1_ and *X*_2_ by using the average value of the real variables, noted ${\bar{X}}_{i}$, *i* = 1, 2, and the half of the variation domain of the variables, noted $\Delta X_{i}$, *i* = 1, 2, defined by relations (2) and (3):

(2) $${\bar{X}}_{i}=\frac{x_{i\min}+x_{i\max}}{2},\;i=1,\;2$$

(3) $$\Delta X_{i}=\frac{x_{i\max}-x_{i\min}}{2},\;i=1,2.$$

The dimensionless variables depend on the real variables as relation (4) indicates:

(4) $$x_{i}=\frac{X_{i}-{\bar{X}}_{i}}{\Delta X_{i}},\;i=1,\;2.$$

Let us propose a dependence (5) of the reaction yield [31,32,33], *η*, on the dimensionless variables, considering the individual influence and the conjugate effects of these variables:

(5) $$\eta =a_{0}+a_{1}x_{1}+a_{2}x_{2}+a_{12}x_{1}x_{2}+a_{11}x_{1}^{2}+a_{22}x_{2}^{2},\;i=1,\;2.$$

**Figure 1:**
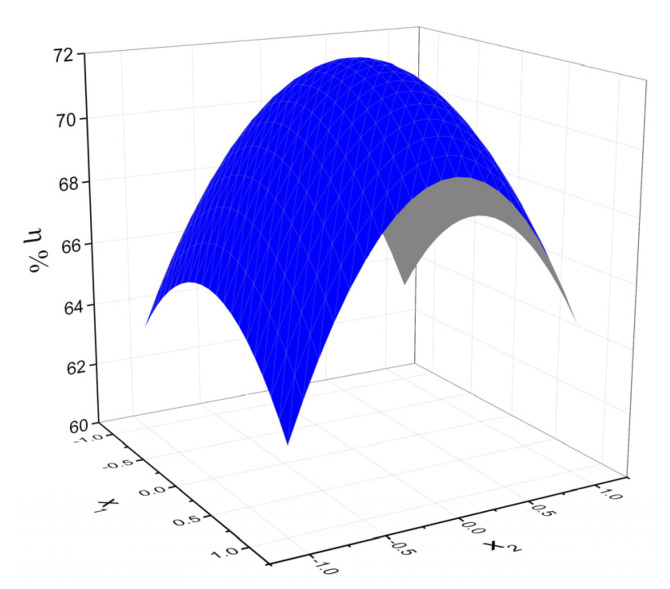
Yield map obtained for molecule VIII.

**Figure 2:**
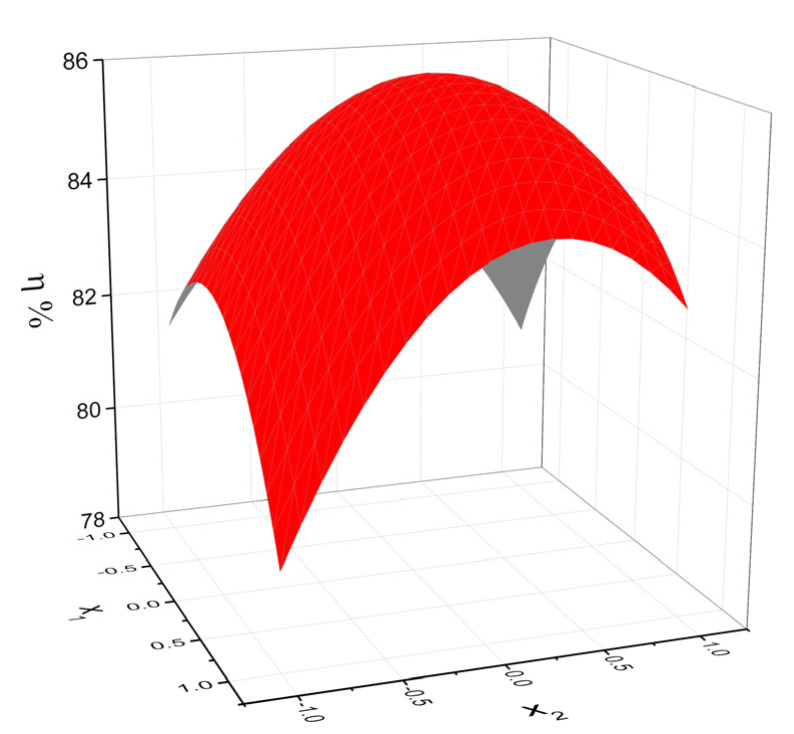
Yield map obtained for molecule IX.

**Figure 3:**
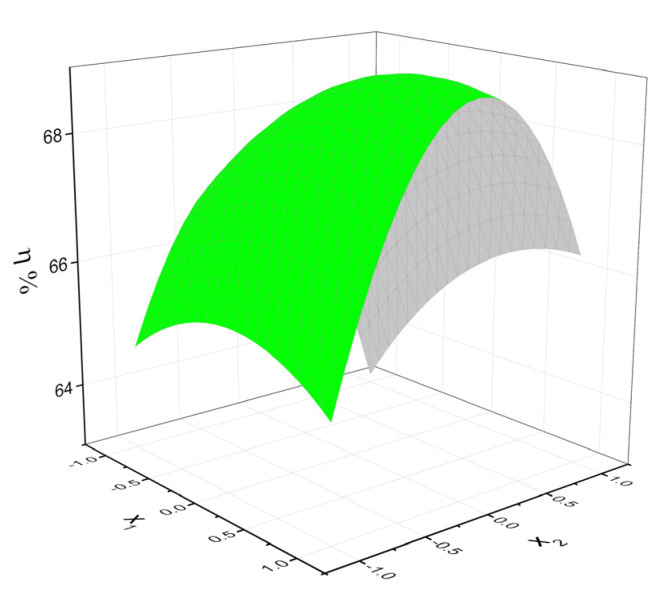
Yield map obtained for molecule IX.

The regression coefficients, *a*_i_, can be determined using the values of *η* and of the dimensionless variables obtained in 3^2^ experiments organized for the chemical reaction for obtaining benzoxazoles. The results are given in Table 1, Table 2 and Table 3 and in Figure 1, Figure 2 and Figure 3 for the molecules VIII, IX and X, respectively.

The absolute values of the coefficients indicate the strength of the dependence of the reaction yield on the corresponding variables. The positive sign of these coefficients indicates the increasing in yield when the corresponding variables increase. The decrease of the reaction yield when the dimensionless variables increase is indicated by the negative sign of the corresponding coefficients.

Using the data from Table 1, Table 2 and Table 3, the regression coefficients from Table 4 were obtained for the three studied compounds.

To decide the situations for which the reaction yield does not depend on a given variable, we must know the *t*-Student coefficients [32]. For this purpose, supplemental experiments were organized in the vicinity of the middle of the variation domain of the dimensionless variables (see Table 5).

The precision of determination of the regression coefficients in relation (5) was estimated by using relation (6):

(6) $$P=\sqrt{\frac{S_{\eta }^{2}}{N}},$$

where

(7) $$S_{\eta }^{2}=\frac{\sum_{1}^{3}{\left(\eta_{i}-\eta \right)}^{2}}{n-1}.$$

In relations (6) and (7) *N* is the number of the experiments in 3^2^ procedure and *n* is the number of the supplemental experiments organized for precision estimation. To compute the sum of quadratic deviations the data of Table 5 were used. The *t*-Student coefficients, estimated on the basis of the values of the regression coefficients from Table 4, are given in Table 6.

If one supposes that the variables corresponding to the *t*-Student coefficients are smaller than 3.00, this does not exert a significant influence on the reaction yield, relation (5) can be written for the studied molecules in relations (8)–(10):

(8) $$\eta_{VIII}=72.22+0.17x_{1}+0.50x_{2}+0.50x_{1}x_{2}-2.84x_{1}^{2}-5.83x_{2}^{2},$$

(9) $$\eta_{IX}=86.11+0.67x_{2}+1.00x_{1}x_{2}-2.17x_{1}^{2}-2.67x_{2}^{2},$$

(10) $$\eta_{X}=68.89+0.83x_{1}+0.75x_{1}x_{2}-0.83x_{1}^{2}-3.33x_{2}^{2}.$$

### 3.4. Quantum Mechanical Analysis of N-Mustards

Since the N-mustards described above are new compounds, the quantum mechanical characterization is necessary because the provided results can help in obtaining information on the interaction mechanism of these molecules with living cells.

**Figure 4:**
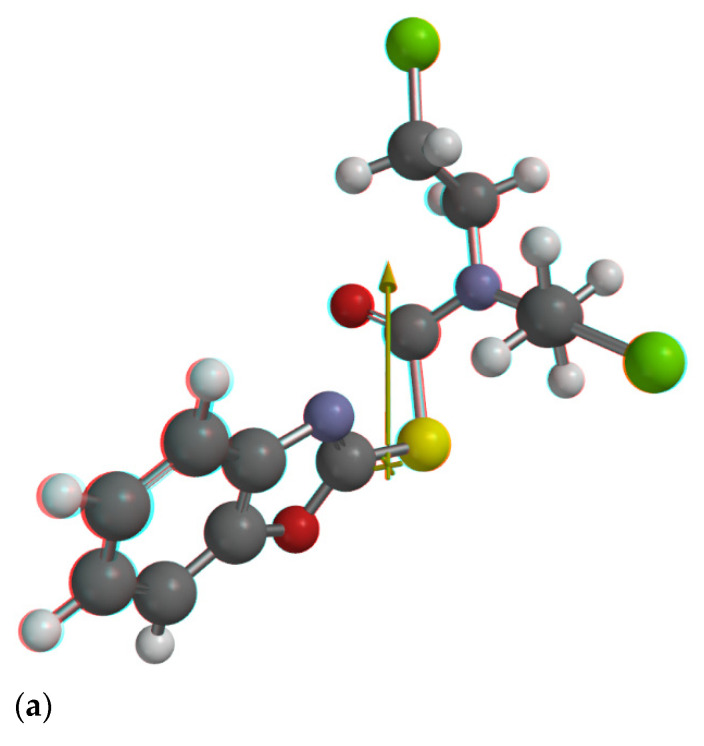

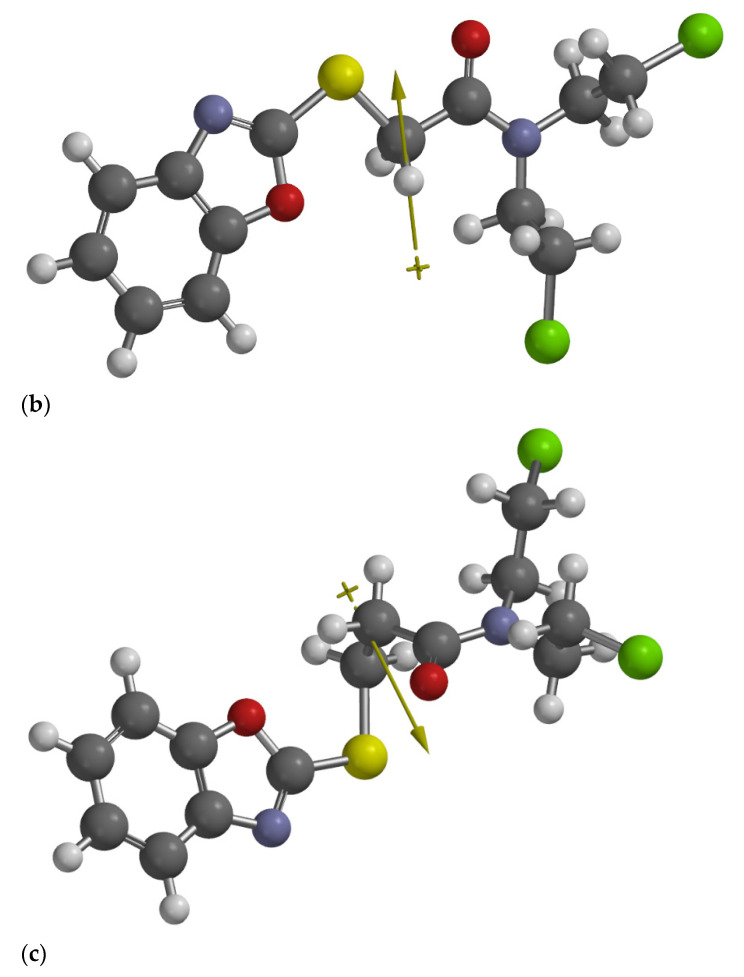
Optimized structure of molecules VIII (**a**), IX (**b**), and X (**c**).

The optimized structures of the three molecules, performed by Spartan’14 software, are given in Figure 4a–c. In Figure 4a–c oxygen atom is red, nitrogen is blue, sulfur is yellow, chlorine is green, carbon is black, and hydrogen is white. Resulting from Figure 4a–c, the reciprocal orientation of the atomic groups in the optimized structure of N-mustards VIII-X and the change in the dipole moment orientation when CH_2_ group is introduced in the structures IX and X. The arrow indicates the orientation of the electrical dipole moment of the molecule.

The values of some electro-optical parameters computed using Spartan’14 for the studied molecules are given in Table 7.

The dipole moment, measuring the separation of the positive and negative electrical charges of the molecule, increases when passing from molecule VIII to molecule X, as results from Table 7. The molecular polarizability, giving information about the induced dipole moment in the molecule by the internal electric field acting in each point in solution, also increases from molecule VIII to molecule X.

The polar surface area (PSA) [47,48,49,50,51] defined as the sum of the polar atoms’ surfaces in a molecule is a useful parameter for prediction of drug transport properties. The values of PSA are between 28.490 and 29.781 Å^2^, these molecules being able to cross both the cell membranes and the blood-brain barrier.

The log P [47,48,49,50,51] value is a measure of lipophilicity or hydrophobicity. The chemical structures of the studied molecules are hydrophobic because the values of logP are positive.

**Figure 5:**
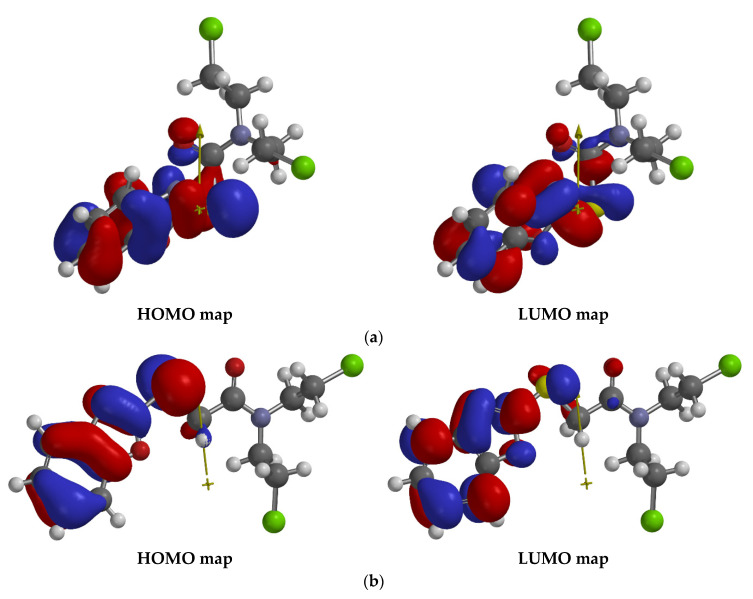

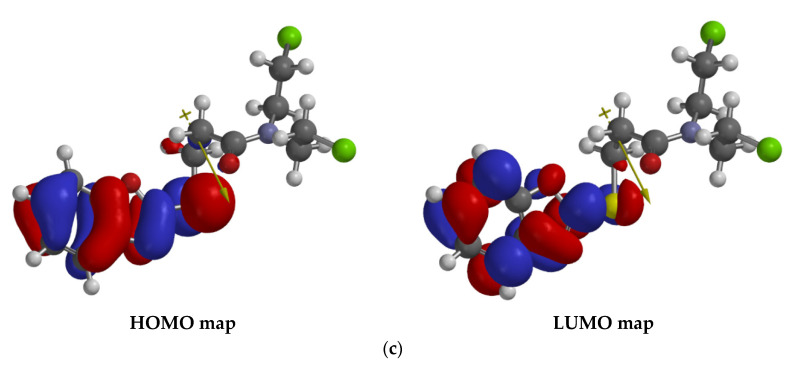
HOMO and LUMO maps of the molecules VIII (**a**), IX (**b**), and X (**c**).

The HOMO and LUMO maps of the studied N-mustards are illustrated in Figure 5a–c. HOMO and LUMO orbitals are involved in the chemical reactivity of the molecule, determining the chemical stability, the electrical and optical properties, and the interaction ability of the molecule with other species.

Figure 5a–c illustrates the changes in the valence electron cloud distribution by excitation; these changes involve especially the heterocycle region. As results from the data of Table 7 referring to the energy gap |ΔE|(eV), the transitions between HOMO and LUMO are made under the UV photons. Figure 5a–c also emphasizes the atomic groups involved in the chemical reactions both in the ground and excited states of N-mustards molecules.

**Figure 6:**
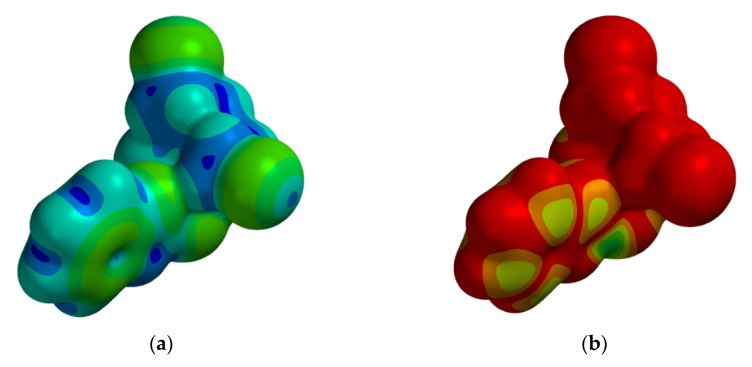
Local ionization map (**a**) and |LUMO| map (**b**) for molecule VIII.

**Figure 7:**
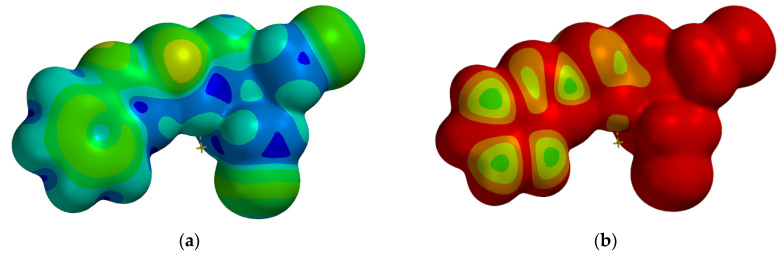
Local ionization map (**a**) and |LUMO| map (**b**) for molecule IX.

**Figure 8:**
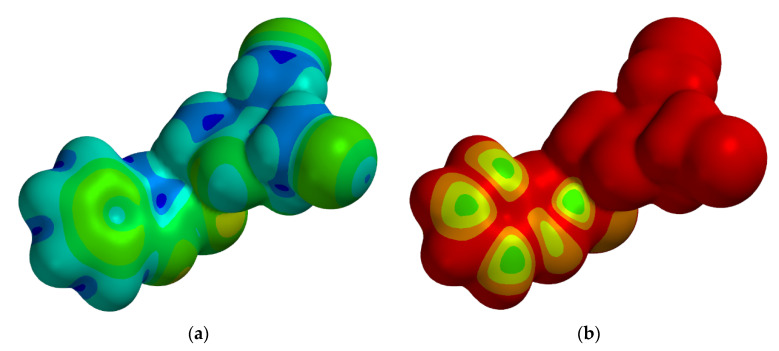
Local ionization map (**a**) and |LUMO| map (**b**) for molecule X.

Some graphical representations provided by Spartan’14 program [34,35] are illustrated in Figure 6, Figure 7 and Figure 8.

Some information can be extracted from the analysis of Figure 6, Figure 7 and Figure 8.

The local ionization potential map (Figure 6a, Figure 7a and Figure 8a) is an indicator of the electrophile addition. Colors in red correspond to small ionization potentials, while colors in blue correspond to high ionization potentials. The studied molecules are characterized by high and intermediate ionization potentials.

|LUMO| map (Figure 6b, Figure 7b and Figure 8b) is an indicator of nucleophilic addition. Colors in red indicate absolute low values of LUMO, while colors in blue indicate absolute high values of LUMO. For molecules VIII-X, the absolute values of LUMO are close to zero.

**Figure 9:**
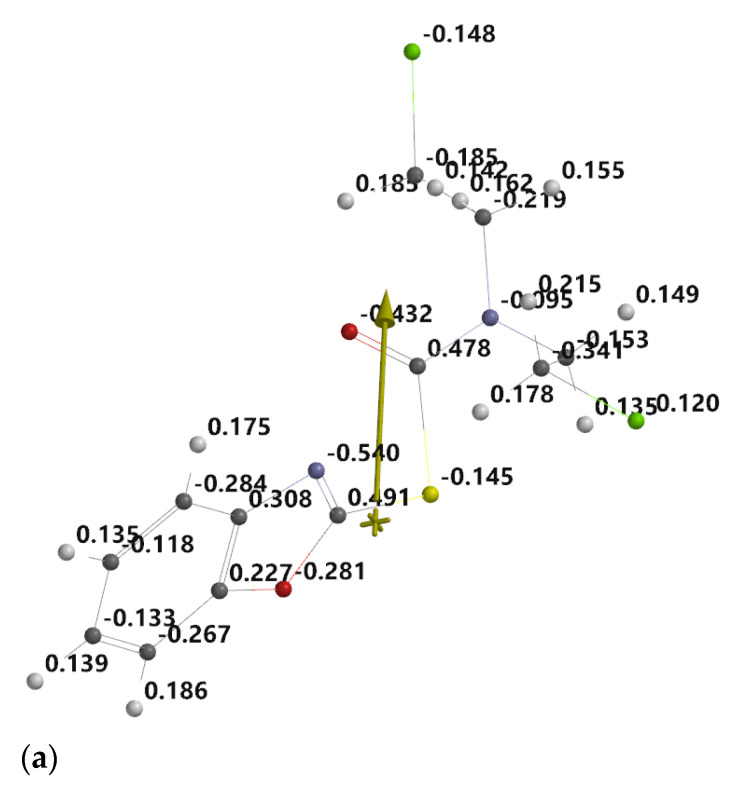

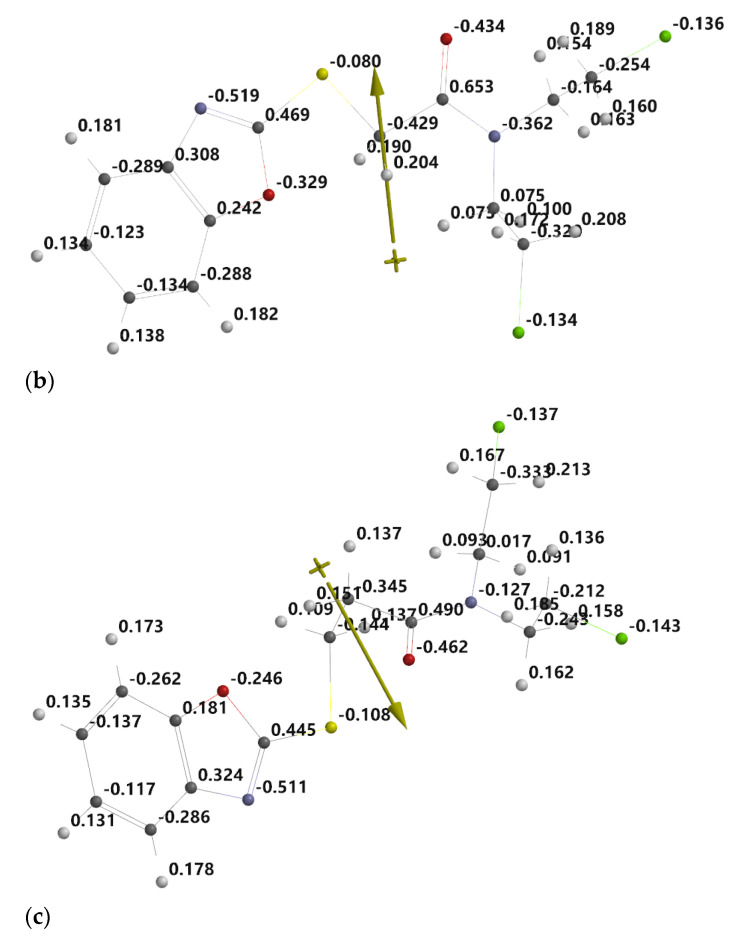
Electrostatic charges near the atoms composing the structures of molecules VIII (**a**), IX (**b**), and X (**c**), respectively.

Figure 9a–c shows the electrostatic charges near the constituent atoms of the studied molecules, calculated using Spartan’14 program and expressed as a percentage of the elementary electric charge.

From Figure 9a–c, it results that the highest negative charges are localized near nitrogen atom belonging to the heterocycle and oxygen atom of the C = O group in all studied molecules.

The arrows in Figure 9a–c show the orientation of the electric dipole moment in the ground electronic states of the studied molecules. It is parallel to the CS bond for the molecule VIII and makes angles near 40–45 degrees with this bond in the other two molecules.

### 3.5. Biological Properties

#### 3.5.1. Assessment of Toxicity of Acids II–IV and N-Mustards VIII–X Compared to Di-(β-Chloroethyl)-Amine

The mice weighing 20 ± 2 g were used and the experimental groups consisted of 10 animals of both sexes. The substances were administered intraperitoneally as a suspension in Twen 80 and the mortality was recorded at 24 h, 48 h, and 7 days. The degree of toxicity (LD_50_) was determined using the Spearman–Kärber method [36]. The obtained results are listed in Table 8.

It is found that the N-mustards (VIII–X) present a very low toxicity compared to that of the di-(β-chloroethyl)-amine free base and a slightly increased toxicity compared to the support intermediates (II–IV). The results confirm the data from the literature according to which the use of heterocyclic structures as transport agents positively influences the decrease of cytotoxic group toxicity [1,52,53,54].

Thus, in the case of N-mustards, the toxicity is due to the support, which causes a decrease in the basicity of nitrogen atom by involvement in the amide conjugation system of its non-participating electrons.

#### 3.5.2. Antitumor Activity of di-(β-Chloroethyl)-Amides VIII-X

In order to study the cytostatic action, the A_2_G mice weighing 25–30 g (±2 g) as experimental animals were used, and *Ascita Ehrlich* was used as an experimental tumor. The transplantation was performed intraperitoneally (10^6^ cells dose). The substances were administered as suspensions in 1% methylcellulose, by single injections on the 7th day after transplantation. The concentrations of 400, 200, and 40 mg/kg body weight were used. The experimental groups included 20 animals for each concentration and the control groups had 10 tumored animals. The inhibition exerted by N-mustards VIII–X was calculated according to the methods from the literature [37], 7 days after the administration of the substances (Table 9), with an error of 1%.

From the experimental data it results:

- The tested N mustards presents appreciable antitumor activity; more selective is the N mustard IX: di-(β-chloroethyl)-amide of benzoxazole-2-yl-mercapto-acetic acid which inhibits *Ehrlich’s Ascites* in a proportion close to that of the reference cytostatic, I.O.B.-82;
- In addition to the alkylating effect, the synthesized N-mustards may also have a specific antimetabolite effect;
- The selective transport of the di-(β-chloroethyl)-amine group performed by the benzoxazole heterocycle and the active group transforms it into a masked group (protected group);
- The relationship between the benzoxazole nucleus and the active group does not consist exclusively in transport, but also in their mutual potentiation (convenient or not, direct or indirect).

## 4. Conclusions

The paper presents results related to the extension of the grafting reaction of the di-(β-chloroethyl)-amine group on heterocyclic structures.

The technique of obtaining di-(β-chloroethyl)-amides with benzoxazole structure was applied for the first time using the activation of support acids derived from 2-mercaptobenzoxazole by transforming them into mixed pivalic anhydrides which, for electronic and steric reasons, can be cleaved by the aminic nitrogen to one of the two carbonyls in the desired direction.

The synthesized products were characterized by elemental and spectral analysis (IR, ^1^H-NMR).

The new compounds VIII –X were characterized by Spartan’14 and their electro-optical properties were established.

The importance of the synthesized N mustards among the biologically active products was highlighted by establishing the degree of toxicity and testing their antitumor activity on experimental animals.

## Figures and Tables

**Table 1:** Experiment 3^2^ for molecule VIII.

No.	*x*_1_ (*X*_1_, °C)	*x*_2_ (*X*_2_, h)	*x*_1_ *x*_2_	$x_{1}^{2}-2/3$	$x_{2}^{2}-2/3$	*η* (%)
1	−1 (65)	−1 (2.5)	1	1/3	1/3	64
2	−1 (65)	0 (2.5)	0	1/3	−2/3	70
3	−1 (65)	1 (2.5)	−1	1/3	1/3	65
4	0 (70)	−1(3.0)	1	−2/3	1/3	68
5	0 (70)	0 (3.0)	0	−2/3	−2/3	73
6	0 (70)	1 (3.0)	−1	−2/3	1/3	67
7	1 (75)	−1 (3.5)	1	1/3	1/3	63
8	1 (75)	0 (3.5)	0	1.3	−2/3	71
9	1 (75)	1 (3.5)	−1	1/3	1/3	66
Σ	0	0	0	0	0	607

**Table 2:** **E**xperiment 3^2^ for molecule IX.

No.	*x*_1_ (*X*_1_, °C)	*x*_2_ (*X*_2_, h)	*x*_1_ *x*_2_	$x_{1}^{2}-2/3$	$x_{2}^{2}-2/3$	*η* (%)
1	−1 (65)	−1 (2.5)	1	1/3	1/3	82
2	−1 (65)	0 (2.5)	0	1/3	-2/3	83
3	−1 (65)	1 (2.5)	−1	1/3	1/3	81
4	0 (70)	−1(3.0)	1	−2/3	1/3	82
5	0 (70)	0 (3.0)	0	−2/3	−2/3	87
6	0 (70)	1 (3.0)	−1	−2/3	1/3	84
7	1 (75)	−1 (3.5)	1	1/3	1/3	80
8	1 (75)	0 (3.5)	0	1.3	−2/3	84
9	1 (75)	1 (3.5)	−1	1/3	1/3	83
Σ	0	0	0	0	0	746

**Table 3:** **E**xperiment 3^2^ for molecule X.

No.	*x*_1_ (*X*_1_, °C)	*x*_2_ (*X*_2_, h)	*x*_1_ *x*_2_	$x_{1}^{2}-2/3$	$x_{2}^{2}-2/3$	*η* (%)
1	−1 (65)	−1 (2.5)	1	1/3	1/3	64
2	−1 (65)	0 (2.5)	0	1/3	−2/3	68
3	−1 (65)	1 (2.5)	−1	1/3	1/3	63
4	0 (70)	−1(3.0)	1	−2/3	1/3	65
5	0 (70)	0 (3.0)	0	−2/3	−2/3	69
6	0 (70)	1 (3.0)	−1	−2/3	1/3	66
7	1 (75)	−1 (3.5)	1	1/3	1/3	65
8	1 (75)	0 (3.5)	0	1.3	−2/3	68
9	1 (75)	1 (3.5)	−1	1/3	1/3	67
Σ	0	0	0	0	0	595

**Table 4:** Regression coefficients for the analyzed molecules.

Molecule	*a*_0_	*a*_1_	*a*_2_	*a*_12_	*a*_11_	*a*_22_
VIII	72.218	0.167	0.5	0.5	−2.839	−5.833
IX	86.111	0.167	0.667	1	−2.166	−2.667
X	68.888	0.830	0.333	0.75	−0.833	−3.333

**Table 5:** Yields for the experiments in the middle of the variation domain.

Molecule	*η*_1_	*η*_2_	*η*_3_	*η*
VIII	71.0	73.5	74.0	72.83
IX	86.11	85.80	85.70	85.87
X	69.3	68.1	69.4	68.93

**Table 6:** *t*-Student coefficients.

Molecule	*t*_0_	*t*_1_	*t*_2_	*t*_12_	*t*_11_	*t*_22_
VIII	830.14	18.93	5.67	5.67	32.13	66.13
IX	1209.42	2.35	9.37	14.04	30.42	37.46
X	285.67	3.46	1.38	3.11	3.46	13.83

**Table 7:** Molecular parameters computed by Spartan’14.

No.	Parameter	Molecule VIII	Molecule IX	Molecule X
1	Solvation energy (kcal/mol)	−35.60	−53.64	−61.09
2	*E*_HOMO_ (eV)	−6.28	−5.94	−5.90
3	*E*_LUMO_ (eV)	−1.29	−0.84	−0.77
4	|ΔE|(eV)	4.99	5.10	4.13
5	Dipole moment (D)	1.53	4.40	4.33
6	Polarizability (Å^3^)	62.65	64.07	65.57
7	Area (Å^2^)	304.38	324.54	342.11
8	Volume (Å^3^)	276.89	294.66	313.21
9	PSA (Å^2^)	29.781	28.490	28.650
10	Log P	2.26	1.49	1.78
11	Ovality	1.48	1.52	1.53
12	Molecular radius (Å)	2.7291	2.7238	2.7466
13	R^3^ (Å^3^)	20.3253	20.2080	20.7192

**Table 8:** The LD50 values (mg/kg body weight) for the compounds II–IV, VIII–X.

Compound	LD_50_			
	24 H	48 H	7 Days	Average
II	1695	1695	1682	1690
III	1830	1830	1790	1816
IV	1725	1725	1690	1713
VIII	1620	1620	1595	1611
IX	1790	1790	1740	1773
X	1685	1685	1650	1673
Di-(β-chloroethyl)-amine	378			

**Table 9:** Inhibition (%) of N mustards VIII–X.

The Tested Compound	Experimental Animals	Route of Administration	Inhibition (%)		
			*Ehrlich Ascites*		
			mg/kg Body Weight		
			400	200	40
VIII	mice	i.p.	43	30	24
IX	mice	i.p.	74	66	53
X	mice	i.p.	52	46	39
I.O.B-82	mice	i.p.	88	79	68

## Data Availability

The data presented in this study are available on request from the corresponding author.
